# Identification of Therapeutic Targets and Prognostic Biomarkers Among CXC Chemokines in the Renal Cell Carcinoma Microenvironment

**DOI:** 10.3389/fonc.2019.01555

**Published:** 2020-02-05

**Authors:** Qingquan Zeng, Shuolei Sun, Yaxian Li, Xiaoling Li, Zuwei Li, Hao Liang

**Affiliations:** ^1^Department of Nephrology, Maoming People's Hospital, Maoming, China; ^2^Department of Urology, Peking University Shenzhen Hospital, Shenzhen, China; ^3^Department of Urology, Maoming People's Hospital, Maoming, China; ^4^Department of Nephrology, Maonan People's Hospital, Maoming, China; ^5^Department of Urology, Gaozhou People's Hospital, Maoming, China; ^6^Department of Hepatology, Gaozhou People's Hospital, Maoming, China

**Keywords:** renal cell carcinoma, biomarker, bioinformatics analysis, tumor microenvironment, chemokine

## Abstract

**Background:** Renal cell carcinoma (RCC) is one of the most common malignances with an ever-increasing incidence and high mortality. Cross-talk between cancer cells and interstitial cells exerts significant effects on neoplasia and tumor development and is modulated in part by chemokines. CXC chemokines in the tumor microenvironment can modulate immune cell trafficking and regulate tumor cell activities, thus exerting anti-tumor immunological effects and affecting patient outcomes; however, the expression and prognostic values of CXC chemokines in RCC have not been clarified.

**Methods:** ONCOMINE, GEPIA, UALCAN, cBioPortal, GeneMANIA, DAVID 6.8, Metascape, TRRUST, LinkedOmics, and TIMER were utilized in this study.

**Results:** The transcriptional levels of *CXCL1/2/5/6/9/10/11/16* in RCC tissues were significantly elevated while the transcriptional levels of CXCL3/7/12/13 were significantly reduced. A significant correlation was found between the expression of *CXC1/5/9/10/11/13* and the pathological stage of RCC patients. RCC patients with low transcriptional levels of *CXCL1/2/3/5/13* were associated with a significantly better prognosis. The functions of differentially expressed CXC chemokines are primarily related to the chemokine signaling pathway, cytokine–cytokine receptor interactions, and the ILK signaling pathway. Our data suggest that RELA, NFKB1, and SP1 are key transcription factors for CXC chemokines, and the SRC family of tyrosine kinases (LCK, LYN, and FYN), mitogen-activated protein kinases (MAPK1 and MAPK3), and CSNK1D are CXC chemokine targets. We found significant correlations among the expression of CXC chemokines and the infiltration of six types of immune cells (B cells, CD8^+^ T cells, CD4^+^ T cells, macrophages, neutrophils, and dendritic cells).

**Conclusions:** Our results may provide novel insights for the selection of immunotherapeutic targets and prognostic biomarkers for renal cell carcinoma.

## Introduction

Kidney cancer is one of the most common malignant tumors, with an incidence of over 400,000 and over 175,000 cancer-associated deaths per annum (1). Originating from epithelial cells of the kidney, renal cell carcinoma (RCC) accounts for more than 90% of renal cancers (2). Accumulating evidence demonstrates that the 5-year disease-specific survival of patients with stage I RCC is ~80–95%, while it drops sharply to <10% in patients with stage IV RCC, whose median overall survival is only 10–15 months (3). At present, clinicians rely primarily on the Tumor Node Metastasis system to predict (with certain limitations) the clinical outcome of patients with RCC. Recently, many researchers have explored the therapeutic targets of RCC, especially kinase and immune checkpoint inhibitors, and some progress has been made (4, 5); however, this is far from sufficient, and more therapeutic targets and prognostic biomarkers must be identified.

Chemokines, a family of approximately 50 low-molecular-weight, chemotactic cytokines, are involved in many biological processes including angiogenesis, tumor growth and metastasis, and the migration of leukocytes (6). Secreted by tumor cells and other cell types, including immune cells, and stromal cells in the tumor microenvironment, chemokines can modulate immune cell trafficking and lymphoid tissue growth, thus regulating anti-tumor immunological responses in a spatiotemporal manner (7, 8). Cumulative evidence has revealed that chemokines can modulate tumor immunity and modulate tumor immunological and biological phenotypes in direct and indirect ways, thus affecting angiogenesis, tumorigenesis, progression, metastasis, therapeutic effect, and patient outcomes (7, 9–11). As an important part of the chemokine family, CXC chemokines are potential therapeutic targets, and prognostic biomarkers for many types of tumors, including RCC (12–14).

Previous studies have characterized a general expression profile and the function of some CXC chemokines in RCC, but identifying suitable CXC chemokines as therapeutic targets and prognostic biomarkers for RCC is still a tremendous problem that urgently needs attention. With the rapid development of second-generation gene sequencing technology and the establishment of various databases, comprehensive analysis of CXC chemokines has become possible. In this study, we conducted an in-depth and comprehensive bioinformatics analysis of the expression of CXC chemokines in RCC and evaluated their potential as therapeutic targets and prognostic biomarkers based on several large public databases, thus providing additional data to help clinicians select appropriate therapeutic drugs and more accurately prognose long-term outcome in patients with RCC.

## Materials and Methods

### ONCOMINE

ONCOMINE (www.oncomine.org) is a translational bioinformatics service that provides powerful, genome-wide expression analysis (15). Data were extracted to evaluate the expression of CXC chemokines in RCC. In this study, a *p* 0.05, a fold change of 2, and a gene rank in the top 10% were set as the significance thresholds. Student's *t* test was used to analyze the difference in the expression of CXC chemokines in RCC.

### GEPIA

GEPIA (http://gepia.cancer-pku.cn/index.html) is an analysis tool containing RNA sequence expression data of 9736 tumors and 8587 normal tissue samples, which was developed at Peking University (16). In this study, we performed a differential mRNA expression analysis of tumor and normal tissues, pathological stage analysis, and correlative prognostic analysis of CXC chemokines with the “Single Gene Analysis” module of GEPIA. Multiple gene comparison analysis of CXC chemokines was performed with the “Multiple Gene Comparison” module of GEPIA, using the “KIRC” dataset. The *p* value cutoff was 0.05. Student's *t* test was used to generate a *p* value for expression or pathological stage analysis. Prognostic analysis was performed using a Kaplan–Meier curve.

### UALCAN

UALCAN (http://ualcan.path.uab.edu/analysis.html), a comprehensive web resource, provides analyses based on The Cancer Genome Atlas (TCGA) and MET500 cohort data (17). In our study, expression data for CXC chemokines was obtained using the “Expression Analysis” module of UALCAN and the “KIRC” dataset. Student's *t* test was used to generate a *p* value. The *p* value cutoff was 0.05.

### cBioPortal

cBioPortal (www.cbioportal.org), a comprehensive web resource, can visualize and analyze multidimensional cancer genomics data (18). Based on TCGA database, genetic alterations, co-expression, and the network module of CXC chemokines was obtained from cBioPortal. Five hundred twelve renal clear cell carcinoma samples (TCGA, provisional) were analyzed. mRNA expression *z* scores (RNA Seq V2 RSEM) were obtained using a *z* score threshold of ±2.0. Protein expression *z* scores (RPPA) were obtained using a *z* score threshold of ±2.0.

### GeneMANIA

GeneMANIA (http://www.genemania.org) is a user-friendly website that provides information for protein and genetic interactions, pathways, co-expression, co-localization, and protein domain similarity of submitted genes (19).

### String

STRING (https://string-db.org/) aims to collect, score, and integrate all publicly available sources of protein–protein interaction (PPI) data, and to complement these with computational predictions of potential functions (20). We conducted a PPI network analysis of differentially expressed CXC chemokines to explore the interactions among them with STRING.

### David 6.8

DAVID 6.8 (https://david.ncifcrf.gov/home.jsp) is a comprehensive, functional annotation website that helps investigators better clarify the biological function of submitted genes (21). In our study, the Gene Ontology (GO) enrichment analysis and Kyoto Encyclopedia of Genes and Genomes (KEGG) pathway enrichment analysis of CXC chemokines and closely related neighbor genes were isolated from DAVID 6.8 and visualized with R project using a “ggplot2” package and a *p* < 0.05. Biological processes (BP), cellular components (CC), and molecular function (MF) were included in the GO enrichment analysis.

### Metascape

Metascape (http://metascape.org) is a reliable, intuitive tool for gene annotation, and gene list enrichment analysis (22). Based on the functional annotation of gene/protein lists, Metascape can facilitate data-driven decisions. In this study, the “Express Analysis” module was used to further verify the enrichment of CXC chemokines and closely related neighbor genes.

### TRRUST

TRRUST (https://www.grnpedia.org/trrust/) is a reliable, intuitive tool for human, and mouse transcriptional regulatory networks. Containing 8444 transcription factor (TF)-target regulatory relationships of 800 human TFs, the TRRUST database can provide information on how these interactions are regulated (23).

### Timer

TIMER (https://cistrome.shinyapps.io/timer/) is a reliable, intuitive tool that provides systematic evaluations of the infiltration of different immune cells and their clinical impact (24). In our study, “Gene module” was used to evaluate the correlation between CXC chemokines level and the infiltration of immune cells. “Survival module” was used to evaluate the correlation among clinical outcome and the infiltration of immune cells and CXC chemokine expression.

### LinkedOmics

LinkedOmics (http://www.linkedomics.org/) is a publicly available portal tool that provides comprehensive multi-omics data analysis across 32 TCGA cancer types (25). We used the “LinkInterpreter” module to derive biological insights into kinase target enrichment, miRNA target enrichment, and transcription factor target enrichment of CXC chemokines. Gene Set Enrichment Analysis (GSEA) was used to perform analyses with a minimum number of genes (size) of 3 and a simulation of 500, within the KIRC dataset. Results were analyzed statistically using the Spearman correlation test. The *p* value cutoff was 0.05.

## Results

### Aberrant Expression of CXC Chemokines in Patients With RCC

**Figure 1:**
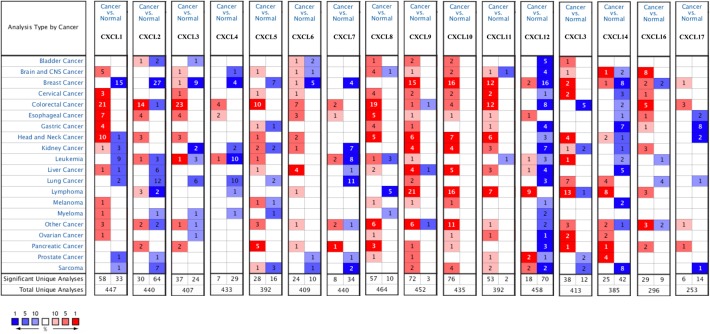
mRNA levels of CXC chemokines in RCC (ONCOMINE). The figure shows the numbers of datasets with statistically significant mRNA over-expression (red) or downregulated expression (blue) of CXC chemokines.

Sixteen CXC chemokines (not including CXCL15) were retrieved using the ONCOMINE database. We first explored the transcriptional levels of CXC chemokines in RCC and normal renal tissues with ONCOMINE. The results are presented in Figure 1 and Table 1. Based on the data from ONCOMINE, the transcriptional levels of *CXCL6, CXCL9, CXCL10, CXCL11*, and *CXCL16* in RCC tissues were significantly elevated while the transcriptional levels of *CXCL3, CXCL7*, and *CXCL13* were significantly reduced in RCC vs. normal renal tissue. These data are consistent with Jones et al. who found a significant downregulation of CXCL3 in RCC (26). Yusenko et al. also revealed that the level of CXCL6 (*p* = 4.80e−4) in RCC was significantly elevated with a fold change of 30.664 (27). Yusenko et al. (27) found a decreased level of CXCL7 in clear cell RCC. Four datasets suggested that CXCL9 expression was elevated in clear cell RCC compared with that in renal tissues (27–30). The transcriptional levels of *CXCL10* in clear cell RCC were remarkably higher than in normal renal tissues in the Yusenko (fold change = 12.873 and *p* = 3.10e−12) and Gumz (fold change = 5.447 and *p* = 5.90e−8) datasets (27, 30). Similarly, Beroukhim's dataset suggested that CXCL10 was significantly upregulated in clear cell RCC (29). The fold change of CXCL10 expression in clear cell RCC was 2.994 (*p* = 9.61e−9), 6.303(*p* = 1.26e−4), and 20.961(*p* = 8.45e−4), in the datasets of Beroukhim (29), Gumz (30), and Yusenko (27), respectively. The results of Yusenko (27), Higgins (31), and Beroukhim (29) all suggested that CXCL13 decreased significantly in RCC tumors compared with normal samples. Moreover, significantly increased levels of CXCL16 were found in RCC tissues (27, 28).

**Table 1:** The mRNA levels of CXC chemokines in different types of RCC tissues and normal renal tissues at transcriptome level (ONCOMINE).

**TLR**	**Type**	**Fold change**	***P*-value**	***t*-test**	**References**
CXCL3	Clear Cell Renal Cell Carcinoma	−2.244	3.88E-7	−8.927	(26)
CXCL6	Clear Cell Renal Cell Carcinoma	30.664	4.80E-4	4.888	(27)
CXCL7	Clear Cell Renal Cell Carcinoma	−9.410	0.009	−3.343	(27)
CXCL9	Clear Cell Renal Cell Carcinoma	2.997	1.41E-6	7.311	(28)
	Clear Cell Renal Cell Carcinoma	31.985	1.81E-7	10.220	(27)
	Clear Cell Renal Cell Carcinoma	4.648	1.26E-5	6.703	(30)
	Clear Cell Renal Cell Carcinoma	7.115	2.22E-7	7.013	(29)
CXCL10	Clear Cell Renal Cell Carcinoma	12.873	3.10E-12	11.075	(27)
	Clear Cell Renal Cell Carcinoma	5.447	5.90E-8	9.505	(30)
	Hereditary Clear Cell Renal Cell Carcinoma	11.612	9.94E-11	9.867	(29)
	Non-Hereditary Clear Cell Renal Cell Carcinoma	5.897	4.41E-7	6.000	(29)
CXCL11	Hereditary Clear Cell Renal Cell Carcinoma	2.994	9.61E-9	7.199	(29)
	Clear Cell Renal Cell Carcinoma	6.303	1.26E-4	5.000	(30)
	Clear Cell Renal Cell Carcinoma	20.691	8.45E-4	5.712	(27)
CXCL13	Clear Cell Renal Cell Carcinoma	−2.697	0.001	−3.676	(27)
	Hereditary Clear Cell Renal Cell Carcinoma	−3.282	9.01E-6	−5.704	(29)
CXCL16	Clear Cell Renal Cell Carcinoma	5.797	5.82E-4	6.812	(27)
	Clear Cell Renal Cell Carcinoma	2.212	7.86E-4	3.932	(28)

**Figure 2:**
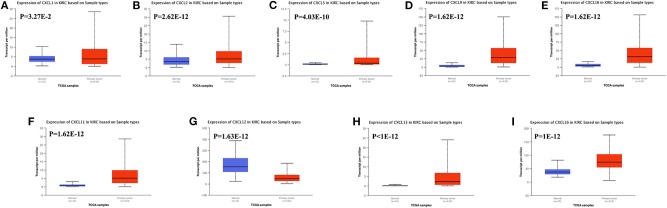
The transcription of CXC chemokines in RCC (UALCAN). The transcriptional levels of **(A)** CXCL1, **(B)** CXCL2, **(C)** CXCL5, **(D)** CXCL9, **(E)** CXCL10, **(F)** CXCL11, **(H)** CXCL13, and **(I)** CXCL16 in RCC tissues were significantly elevated while the transcriptional levels of **(G)** CXCL12 were significantly reduced. The *p* value was set at 0.05.

**Figure 3:**
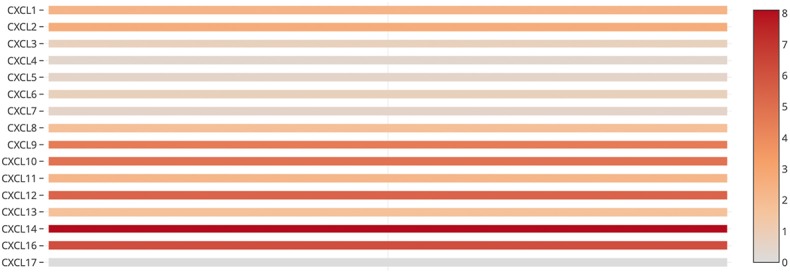
The relative level of CXC chemokines in RCC.

We also assessed the expression levels of CXC chemokines in RCC tumors and normal tissues with UALCAN. As expected, the transcriptional levels of *CXCL1* (*p* = 3.27e−2), *CXCL2* (*p* = 2.62e−12), *CXCL5* (*p* = 4.03e−10), *CXCL9* (*p* = 1.62e−12), *CXCL10* (*p* = 1.62e−12), *CXCL11* (*p* = 1.62e−12), and *CXCL16* (*p* = 1e−12) in RCC tissues were significantly elevated while the transcriptional levels of *CXCL12* (*p* = 1.63e−12) were significantly reduced (Figure 2). We also compared the relative expression levels of CXC chemokines in RCC tissues and found that among all CXC chemokines we evaluated, the relative expression of CXCL14 was the highest (Figure 3). To identify additional CXC chemokines associated with tumorigenesis, progression, and clinical outcome in RCC, we evaluated all the CXC cytokines that were differentially expressed in RCC tumors vs normal tissues (CXCL1, CXCL2, CXCL3, CXCL5, CXCL6, CXCL7, CXCL9, CXCL10, CXCL11, CXCL12, CXCL13, and CXCL16). We excluded CXCL4, CXCL8, CXCL14, and CXCL17 from further analysis since they were expressed at similar levels in RCC tumors and normal tissue.

**Figure 4:**
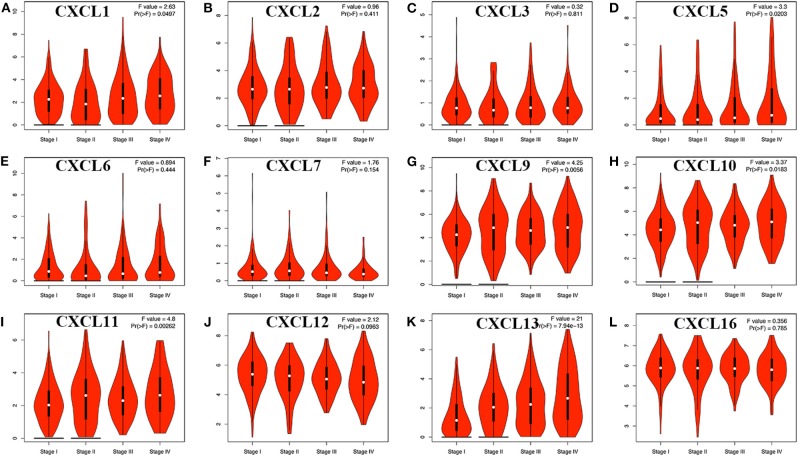
Correlation between different expressed CXC chemokines and the pathological stage of RCC patients (GEPIA). *P < 0.05. **(A)** CXCL1, **(B)** CXCL2, **(C)** CXCL3, **(D)** CXCL5, **(E)** CXCL6, **(F)** CXCL7, **(G)** CXCL9, **(H)** CXCL10, **(I)** CXCL11, **(J)** CXCL12, **(K)** CXCL13, and **(L)** CXCL16.

We then assessed the correlation between the expression of differentially expressed CXC chemokines and the pathological stage of RCC patients and found a significant correlation between the expression of CXC1 (*p* = 0.050), CXC5 (*p* = 0.020), CXC9 (*p* = 0.006), CXC10 (*p* = 0.018), CXC11 (*p* = 0.003), CXC13 (*p* = 7.94e−13), and pathological stage (Figure 4). As the tumor progressed, the expression of CXC1, CXC5, CXC9, CXC10, CXC11, and CXC13 increased. These data suggest that these CXC chemokines play a significant role in the tumorigenesis and progression of RCC.

### The Prognostic Value of CXC Chemokines in Patients With RCC

**Figure 5:**
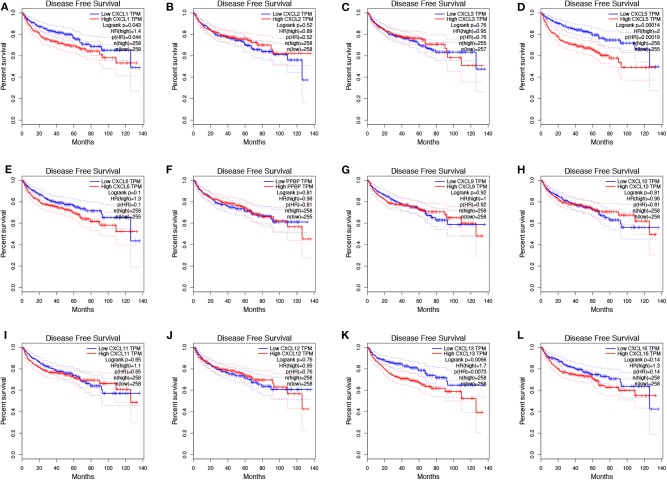
The prognostic value of different expressed CXC chemokines in RCC patients in the disease free survival curve (GEPIA). The disease free survival curve of **(A)** CXCL1, **(B)** CXCL2, **(C)** CXCL3, **(D)** CXCL5, **(E)** CXCL6, **(F)** CXCL7, **(G)** CXCL9, **(H)** CXCL10, **(I)** CXCL11, **(J)** CXCL12, **(K)** CXCL13, and **(L)** CXCL16 in RCC.

**Figure 6:**
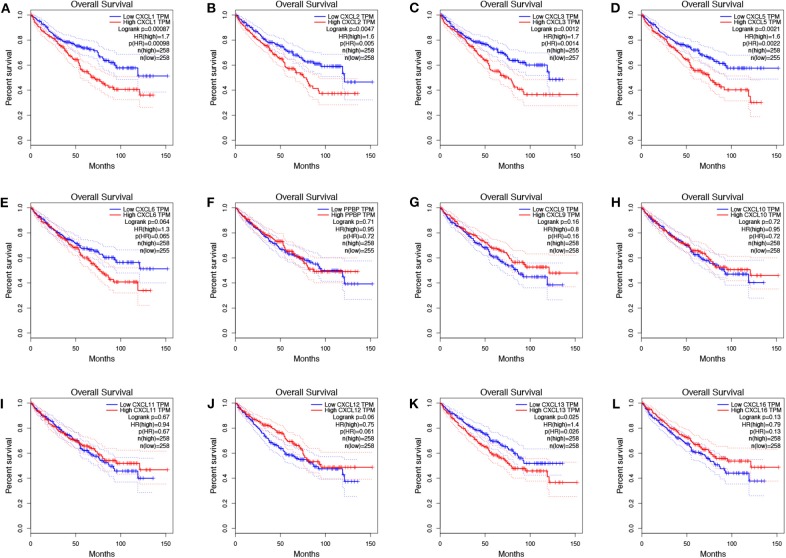
The prognostic value of CXC chemokines in RCC patients in the overall survival curve (GEPIA). The overall survival curve of **(A)** CXCL1, **(B)** CXCL2, **(C)** CXCL3, **(D)** CXCL5, **(E)** CXCL6, **(F)** CXCL7, **(G)** CXCL9, **(H)** CXCL10, **(I)** CXCL11, **(J)** CXCL12, **(K)** CXCL13, and **(L)** CXCL16 in RCC.

To evaluate the value of differentially expressed CXC chemokines in the progression of RCC, we assessed the correlation between differentially expressed CXC chemokines and clinical outcome using GEPIA. Disease-free survival curves are presented in Figure 5. RCC patients with low transcriptional levels of *CXCL1* (*p* = 0.043) and *CXCL5* (*p* = 0.00014) were significantly associated with longer disease-free survival. The value of differentially expressed CXC chemokines in the overall survival of RCC patients was also evaluated. We found that RCC patients with low transcriptional levels of *CXCL1* (*p* = 0.00087), *CXCL2* (*p* = 0.0047), *CXCL3* (*p* = 0.0012), *CXCL5* (*p* = 0.021), and *CXCL13* (*p* = 0.025) were significantly associated with longer overall survival (Figure 6).

### Genetic Alteration, Co-expression, Neighbor Gene Network, and Interaction Analyses of CXC Chemokines in Patients With RCC

**Figure 7:**
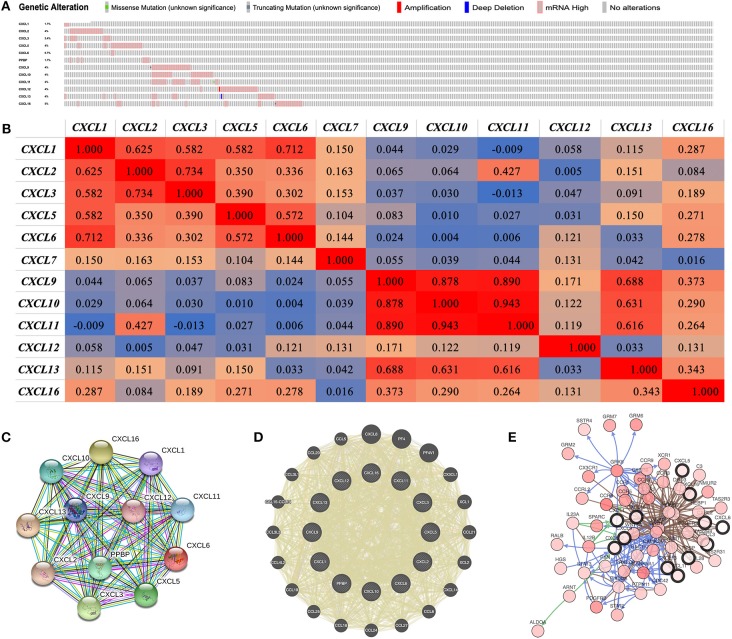
Genetic alteration, neighbor gene network, and interaction analyses of different expressed CXC chemokines in RCC patients. **(A)** Summary of alterations in different expressed CXC chemokines in RCC. **(B)** Correlation heat map of different expressed CXC chemokines in RCC. **(C,D)** Protein–protein interaction network of different expressed CXC chemokines. **(E)** Gene–gene interaction network of different expressed CXC chemokines and 50 most frequently altered neighboring genes.

We performed a comprehensive analysis of the molecular characteristics of differentially expressed CXC chemokines. Provisional datasets of TCGA were utilized to analyze the genetic alterations of differentially expressed CXC chemokines. As a result, *CXCL1, CXCL2, CXCL3, CXCL5, CXCL6, CXCL7, CXCL9, CXCL10, CXCL11, CXCL12, CXCL13*, and *CXCL16* were altered in 1.7, 4, 2.4, 4, 0.7, 1.7, 4, 4, 4, 4, 4, and 5% of the queried RCC samples, respectively (Figure 7A). Enhanced mRNA expression was the most common change in these samples. We next explored the potential co-expression of the differentially expressed CXC chemokines. There was a moderate to high correlation among the expression of *CXCL1, CXCL2, CXCL3*, and *CXCL5* (Figure 7B), a high correlation among *CXCL9, CXCL10*, and *CXCL11* (Figure 6B), and a low to moderate correlation among *CXCL12, CXCL13*, and *CXCL16* (Figure 6B). Moreover, we conducted a PPI network analysis of differentially expressed CXC chemokines with STRING to explore the potential interactions among them. As expected, several nodes of 12 and several edges of 66 were obtained in the PPI network (Figure 7C). The function of these differentially expressed CXC chemokines was associated with the chemokine signaling pathway and the inflammatory response. Results of GeneMANIA also revealed that the functions of differential expressed CXC chemokines (*CXCL1, CXCL2, CXCL3, CXCL5, CXCL6, CXCL7, CXCL9, CXCL10, CXCL11, CXCL12, CXCL13*, and *CXCL16*) were primarily related to cell chemotaxis, chemokine receptor binding, and chemokine activity (Figure 7D). Moreover, the top 50 most frequently altered neighbor genes associated with differentially expressed CXC chemokines were isolated with cBioPortal. These data suggest that *ALDOA, ARNT, C3, CALM1, CAMK2A, CCL28, CCR1, CCR2, CCR3, CCR4, CCR5, CCR8, CCR9, CCRL2, CDC42, CSNK1A1, CX3CR1, CXCR6, DRD3, GNAI2, GNB1, GNB2, GNGT1, GRK6, GRM6, GRM7, HEBP1, HGS, 1L12B, 1L23A, NMUR2, PDGFRB, PIK3CA, PPKCD, PTGER3, PTPN11, PXN, RAC1, RALB, SPARC, SSTR4, STAT2, STAT3, TAS2R3, TAS2R31, TAS2R4, VWF, WNT5A, XCR1*, and *GRM2* were primarily associated with the modulation and function of differentially expressed CXC chemokines in RCC (Figure 7E).

### Functional Enrichment Analysis of CXC Chemokines in Patients With RCC

**Figure 8:**
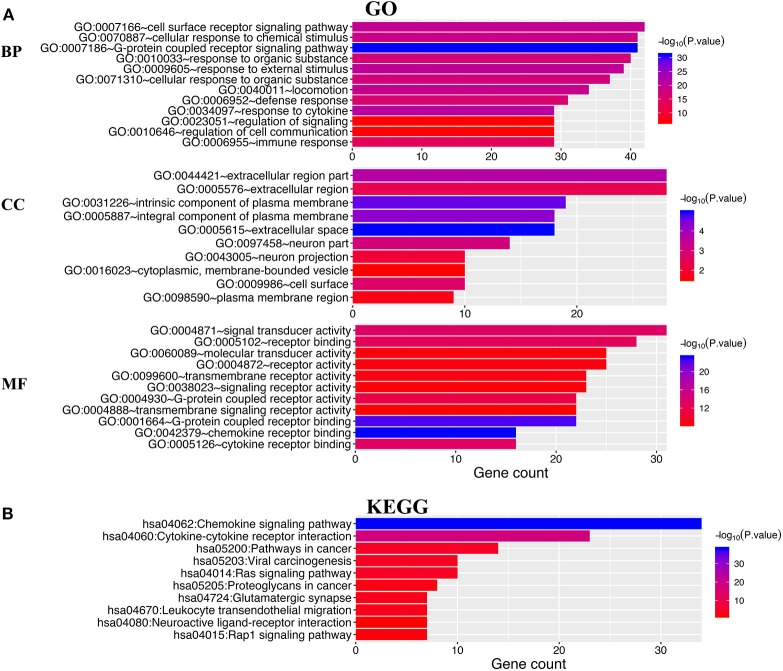
The enrichment analysis of different expressed CXC chemokines and 50 most frequently altered neighboring genes in RCC (David 6.8). **(A)** Bar plot of GO enrichment in cellular component terms, biological process terms, and molecular function terms. **(B)** Bar plot of KEGG enriched terms.

DAVID 6.8 and Metascape were utilized to analyze the functions of differentially expressed CXC chemokines and their neighboring genes. Figure 8A shows the top 10 most highly enriched GO items using DAVID 6.8. Among the 10 most highly enriched functions in the BP category, cell surface receptor signaling pathways, G-protein coupled receptor signaling pathways, defense responses, response to cytokines, and immune responses were associated with the tumorigenesis and progression of RCC. The extracellular region, intrinsic component of the plasma membrane, extracellular space, integral component of plasma membrane, neuronal part, cell surface, neuronal projection, cytoplasmic, membrane-bounded vesicle, and the plasma membrane region were the 10 most highly enriched items in the CC category. In the molecular function MF category, the differentially expressed CXC chemokines and their neighboring genes were mainly enriched in chemokine receptor binding and cytokine receptor binding activities. KEGG pathway analyses were also performed. As expected, among the top 10 KEGG pathways, chemokine signaling pathway, cytokine–cytokine receptor interaction, pathways in cancer, viral carcinogenesis, Ras signaling pathway, proteoglycans in cancer, leukocyte transendothelial migration, and the Rap1 signaling pathway were significantly associated with the tumorigenesis and progression of RCC (Figure 8B).

Supplementary Figure 1 shows the results of the functional enrichment analysis obtained from Metascape. As presented in Supplementary Figures 1A,B, the functions of differentially expressed CXC chemokines and their neighboring genes were mainly enriched in chemokine signaling pathway and leukocyte chemotaxis. To better understand the correlation between differentially expressed CXC chemokines and RCC, the PPI network and mCODE components were analyzed. A list of genes is identified in Supplementary Figures 1C,D. We extracted the two most significant mCODE components from the PPI network and found that biological function was mainly associated with G alpha (i) signaling events, GPCR ligand binding, chemokine receptors, chemokine binding, the PID EPHB FWD pathway, the PID integrin-linked kinase (ILK) pathway, and the positive regulation of stress fiber assembly (Supplementary Figure 1E).

### Transcription Factor Targets, Kinase Targets, and miRNA Targets of STATs in Patients With RCC

Due to the significant difference in the expression of CXC chemokines in RCC vs. normal tissue, we explored possible transcription factor targets and kinase targets of the differentially expressed CXC chemokines using the TRRUST and LinkedOmics databases.

*CXCL1, CXCL2, CXCL5, CXCL7, CXCL10*, and *CXCL12* were included in TRRUST. We found that three transcription factors (RELA, NFKB1, and SP1) were associated with the regulation of CXC chemokines (Table 2). RELA and NFKB1 were the key transcription factors for *CXCL1, CXCL2, CXCL5, CXCL10*, and *CXCL12*. SP1 was the key transcription factor for *CXCL1* and *CXCL5*. We identified the top two kinase targets of CXC chemokines from the LinkedOmics database (results presented in Table 3). Only one kinase target was identified in the CXCL1 (CSNK1D) and the CXCL3 (SGK1) kinase-target network. CSNK1D and FER were the top two targets in the CXCL2 kinase-target network. Components of the CXCL5 kinase-target network were mainly associated with ATM and MAPK1. CDK6 and PRKAA2 were suggested as the targets for the CXCL6 kinase-target network. SRC and MAPK3 were primarily related to CXCL7. ITK and FYN, and ITK and CSNK1E were the top two targets in the CXCL9 and CXCL10 kinase-target networks, respectively. Components of the CXCL11 and CXCL12 kinase-target networks were mainly associated with LCK and FYN, as well as SRC and LYN. LCK and MAPK1 were suggested as targets for the CXCL13 kinase-target network. LYN and FYN were primarily associated with CXCL16.

**Table 2:** Key regulated factor of CXC chemokines in RCC (TRRUST).

**Key TF**	**Description**	**Regulated gene**	***P*-value**	**FDR**
RELA	v-rel reticuloendotheliosis viral oncogene homolog A (avian)	CXCL1, CXCL2, CXCL5, CXCL10, CXCL12	7.21E-07	1.12E-06
NFKB1	nuclear factor of kappa light polypeptide gene enhancer in B-cells 1	CXCL1, CXCL2, CXCL5, CXCL10, CXCL12	7.45E-07	1.12E-06
SP1	Sp1 transcription factor	CXCL1, CXCL5	0.0349	0.0349

**Table 3:** The Kinase target networks of CXC chemokines in RCC (LinkedOmics).

**CXC chemokines**	**Enriched kinase target**	**Description**	**Leading EdgeNum**	***P*-value**
CXCL1	Kinase_CSNK1D	Casein kinase 1 delta	5	0.020
CXCL2	Kinase_CSNK1D	Casein kinase 1 delta	5	0.007
	Kinase_FER	FER tyrosine kinase	3	0.039
CXCL3	Kinase_SGK1	Serum/glucocorticoid regulated kinase 1	1	0.037
CXCL5	Kinase_ATM	ATM serine/threonine kinase	11	0.002
	Kinase _MAPK1	Mitogen-activated protein kinase 1	25	0.007
CXCL6	Kinase_CDK6	Cyclin dependent kinase 6	1	0.005
	Kinase_PRKAA2	Protein kinase AMP-activated catalytic subunit alpha 2	2	0.005
CXCL7	Kinase_SRC	SRC proto-oncogene, non-receptor tyrosine kinase	21	0.007
	Kinase_MAPK3	Mitogen-activated protein kinase 3	8	0.016
CXCL9	Kinase_ITK	IL2 inducible T-cell kinase	2	0.003
	Kinase_FYN	FYN proto-oncogene, Src family tyrosine kinase	7	0.007
CXCL10	Kinase_ITK	IL2 inducible T-cell kinase	2	0.019
	Kinase_CSNK1E	Casein kinase 1 epsilon	4	0.020
CXCL11	Kinase_LCK	LCK proto-oncogene, Src family tyrosine kinase	5	0.017
	Kinase_FYN	FYN proto-oncogene, Src family tyrosine kinase	7	0.012
CXCL12	Kinase_SRC	SRC proto-oncogene, non-receptor tyrosine kinase	21	0
	Kinase_LYN	LYN proto-oncogene, Src family tyrosine kinase	6	0
CXCL13	Kinase_LCK	LCK proto-oncogene, Src family tyrosine kinase	5	0
	Kinase_MAPK1	Mitogen-activated protein kinase 1	12	0
CXCL16	Kinase_LYN	LYN proto-oncogene, Src family tyrosine kinase	6	0
	Kinase_FYN	FYN proto-oncogene, Src family tyrosine kinase	9	0

### Immune Cell Infiltration of CXC Chemokines in Patients With RCC

**Figure 9:**
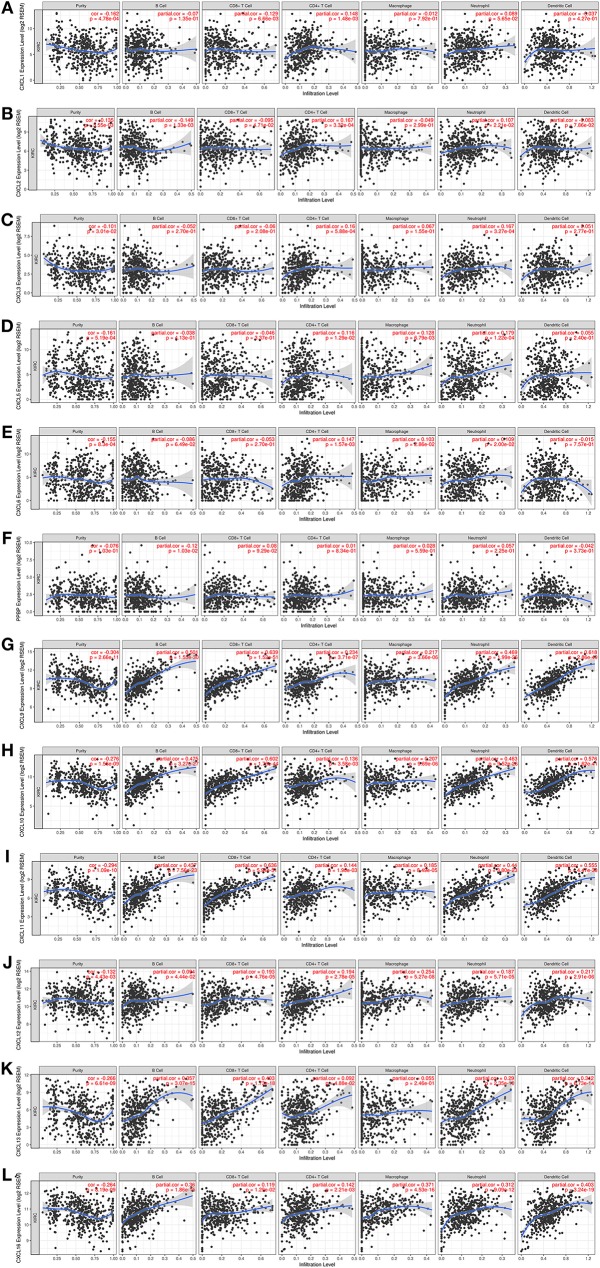
The correlation between different expressed CXC chemokines and immune cell infiltration (TIMER). The correlation between the abundance of immune cell and the expression of **(A)** CXCL1, **(B)** CXCL2, **(C)** CXCL3, **(D)** CXCL5, **(E)** CXCL6, **(F)** CXCL7, **(G)** CXCL9, **(H)** CXCL10, **(I)** CXCL11, **(J)** CXCL12, **(K)** CXCL13, and **(L)** CXCL16 in RCC.

CXC chemokines are involved in inflammatory responses and immune cell infiltration, thus affecting the clinical outcome of RCC patients. Therefore, we embarked on a comprehensive exploration of the correlation between differentially expressed CXC chemokines and immune cell infiltration using the TIMER database. There was a negative correlation between CXCL1 expression and the infiltration of CD8+ T cells (Cor = −0.129, *p* = 6.66e−3), and a positive correlation between CXCL1 expression and the infiltration of CD4^+^ T cells (Cor = 0.148, *p* = 1.48e−3; Figure 9A). CXCL2 expression was negatively associated with the infiltration of B cells (Cor = −0.149, *p* = 1.33e−3) and CD8^+^ T cells (Cor = −0.095, *p* = 4.71e−2), and positively associated with the infiltration of CD4^+^ T cells (Cor = 0.167, *p* = 3.32e−4) and neutrophils (Cor = 0.107, *p* = 2.21e−2; Figure 9B). CXCL3 expression was positively associated with the infiltration of CD4^+^ T cells (Cor = 0.16, *p* = 5.88e−4) and neutrophils (Cor = 0.167, *p* = 3.27e−4; Figure 9C). Similarly, the expression of CXCL5 was positively associated with the infiltration of CD4^+^ T cells (Cor = 0.116, *p* = 1.29e−2), macrophages (Cor = 0.128, *p* = 6.79e−4), and neutrophils (Cor = 0.179, *p* = 1.22e−4; Figure 9D). There was a negative correlation between CXCL6 expression and the infiltration of CD4^+^ T cells (Cor = 0.147, *p* = 1.57e−3), macrophages (Cor = 0.103, *p* = 2.86e−2), and neutrophils (Cor = 0.109, *p* = 2.00e−2; Figure 9E). We also found that the lower the infiltration of B cells, the higher the expression of CXCL7 (Cor = −1.2, *p* = 1.03e−2; Figure 9F). There was a positive correlation between CXCL9 expression and the infiltration of B cells (Cor = 0.501, *p* = 1.55e−30), CD8^+^ T cells (Cor = 0.639, *p* = 1.52e−51), CD4^+^ T cells (Cor = 0.234, *p* = 3.71e−7), macrophages (Cor = 0.217, *p* = 3.66e−6), neutrophils (Cor = 0.469, *p* = 1.99e−26), and dendritic cells (Cor = 0.618, *p* = 2.86e−49; Figure 9G). Similar results were obtained for CXCL10, CXCL11, CXCL12, and CXCL16. There was a positive correlation between the expression of CXCL10, CXCL11, CXCL12, and CXCL16, and the infiltration of B cells, CD8^+^ T cells, CD4^+^ T cells, macrophages, neutrophils, and dendritic cells; all *p* < 0.05; Figures 9H–J,L). Except for macrophages, CXCL13 expression positively correlated with infiltration of the other five immune cell types (B cells, CD8^+^ T cells, CD4^+^ T cells, neutrophils, and dendritic cells; all *p* < 0.05; Figure 9K). We also evaluated correlation of differentially expressed CXC chemokines and immune cell infiltration. The Cox proportional hazard model was used, and we corrected for the following confounding factors: B cells, CD4^+^ T cells, macrophages, neutrophils, CXCL1, CXCL2, CXCL3, CXCL5, CXCL6, CXCL7, CXCL12, and CXCL16. CD8+ T cells (*p* = 0.002), dendritic cells (*p* = 0.016), CXCL9 expression (*p* = 0.048), CXCL10 expression (*p* = 0.046), CXCL11 expression (*p* = 0.004), and CXCL13 expression (*p* = 0) were significantly associated with the clinical outcome of RCC patients (Table 4).

**Table 4:** The cox proportional hazard model of CXC chemokines and six tumor-infiltrating immune cells in RCC (TIMER).

	**coef**	**HR**	**95%CI_l**	**95%CI_u**	***p*-value**	**sig**
B_cell	−2.144	0.117	0.004	3.523	0.217	
CD8_Tcell	−3.065	0.047	0.007	0.318	0.002	^**^
CD4_Tcell	−1.600	0.202	0.011	3.755	0.283	
Macrophage	−1.136	0.321	0.027	3.773	0.366	
Neutrophil	0.367	1.443	0.020	104.796	0.867	
Dendritic	2.415	11.186	1.559	80.279	0.016	^*^
CXCL1	0.131	1.140	0.997	1.302	0.055	
CXCL2	0.015	1.015	0.868	1.187	0.851	
CXCL3	0.039	1.039	0.884	1.222	0.641	
CXCL5	0.021	1.021	0.957	1.091	0.526	
CXCL6	0.006	1.006	0.919	1.101	0.903	
CXCL7	−0.028	0.972	0.870	1.087	0.622	
CXCL9	−0.258	0.772	0.598	0.997	0.048	^*^
CXCL10	−0.340	0.712	0.510	0.994	0.046	^*^
CXCL11	0.508	1.661	1.177	2.345	0.004	^**^
CXCL12	−0.016	0.984	0.862	1.122	0.808	
CXCL13	0.224	1.251	1.140	1.373	0.000	^***^
CXCL16	−0.138	0.871	0.665	1.141	0.317	

* *P < 0.05*,

** *P < 0.01*,

*** *P < 0.001*.

## Discussion

CXC chemokines were initially identified as inflammatory mediators, and they play a significant role in the maturation, differentiation, and trafficking of leukocytes (32). Intercellular communication between RCC cells and stromal cells affects the expression patterns of chemokines in various cell types, thus facilitating specific microenvironments for tumor invasion and metastasis. Accumulating evidence has demonstrated a significant role for CXC chemokines in tumorigenesis, tumor cell proliferation and apoptosis, and tumor metastasis (33, 34). Some studies have reported a correlation among CXC chemokines, the tumor microenvironment, and cancer immunotherapy, suggesting that CXC chemokines may modulate tumor progression and immunotherapeutic effect. However, the prognostic value and biological function of CXC chemokines in RCC have not been well-characterized.

We first explored the expression of CXC chemokines and their correlation with the pathological stage in RCC. We found that 12 genes were differentially expressed in RCC compared with normal tissue (upregulation of *CXCL1, CXCL2, CXCL5, CXCL6, CXCL9, CXCL10, CXCL11, CXCL13*, and *CXCL16*; downregulation of *CXCL3, CXCL7*, and *CXCL12*). Moreover, we found that the expression of *CXC1, CXC5, CXC9, CXC10, CXC11*, and *CXC13* increased as the tumors progressed. RCC patients with low expression of *CXCL1, CXCL2, CXCL3, CXCL5*, and *CXCL13* were significantly associated with better overall survival. These data demonstrate that differentially expressed CXC chemokines may play a significant role in RCC. Gutwein et al. found that CXCL16 expression was significantly enhanced in RCC tissues (12). However, previous studies that studied the expression level and prognostic value of CXC chemokines in various cancers are limited.

Since multiple chemokines were significantly differentially expressed in RCC, we explored their molecular characteristics in RCC. There were frequent genetic alterations in the CXC chemokines differentially expressed in RCC. Elevated mRNA expression was the most alteration. Tumorigenesis and the progression of RCC are complex and multi-faceted, and genetic alteration plays an important role in this process (35). We found a low to high correlation among the differentially expressed CXC chemokines, suggesting that these cytokines play a synergistic role in the tumorigenesis and progression of RCC.

We then focused on the function of differentially expressed CXC chemokines using GO enrichment analysis and KEGG pathway enrichment analysis. As expected, we found that the functions of these genes are primarily related to the chemokine signaling pathway, cytokine–cytokine receptor interactions, and the ILK signaling pathway. Previous studies have demonstrated that chemokine signaling pathways play key roles in the proliferation, senescence, angiogenesis, epithelial–mesenchymal transition, immune evasion, and metastasis of various cancers (36–40). ILK, an ankyrin, repeat-containing serine/threonine protein kinase, plays a significant role in biological processes associated with tumorigenesis, including cancer cell proliferation, angiogenesis, metastasis, and drug resistance (41). These data suggest that the CXC chemokines, which are differentially expressed in RCC, are potential drug therapeutic targets.

We also sought to characterize the transcription factor targets and kinase targets of the differentially expressed CXC chemokines, and found that RELA, NFKB1, and SP1 may be key transcription factors in the regulation of CXC chemokines. RELA phosphorylation is involved in disease progression, notably inflammatory diseases and cancer, by regulating NF-κB signaling (42). Another study demonstrated a key role for RELA in mediating oncogene-induced senescence in preneoplastic lesions (43). NFKB1, a suppressor of inflammation and cancer, plays an inhibitory role in the tumorigenesis and progression of a variety of cancers by reducing the abnormal activation of the NF-κB signaling pathway (44, 45). Moreover, the androgen receptor-induced AKT → NF-κB → CXCL5 signaling pathway drives RCC progression by regulating endothelial cell proliferation and recruitment (46). Our results may provide additional data about the complicated relation among RCC, CXC chemokines, and the NF-κB signaling pathway. Our data indicated that the SRC family tyrosine kinases (LCK, LYN, and FYN), mitogen-activated protein kinases (MAPK1 and MAPK3), and casein kinase 1 delta (CSNK1D) were the probable targets of the differentially expressed CXC chemokines. These kinases are involved in genomic stability, DNA damage, cell cycle progression, and epithelial–mesenchymal transition (47–51). Moreover, these kinases affect tumor development and progression by regulating tumor cell migration, invasion, and apoptosis (52, 53). In RCC, differentially expressed CXC chemokines may modulate genomic stability, DNA repair, cell cycle progression, and the epithelial–mesenchymal transition by regulating these kinases.

Chemokines are chemotactic cytokines mediating the migration and localization of immune cells (54). Increasing evidence suggests that immune cell infiltration could affect tumor progression and recurrence, and act as a significant determinant of both response to immunotherapy and clinical outcome (55, 56). CD4^+^ T cells recognize cancer antigens, and activated M1 macrophages may inhibit cancer growth (57). In this study, we found a significant correlation between the expression of CXC chemokines and the infiltration of the six immune cell types, B cells, CD8^+^ T cells, CD4^+^ T cells, macrophages, neutrophils, and dendritic cells, indicating that CXC chemokines are not only as prognostic indicators, but may also reflect immune status.

Our study has some limitations. Analysis on the transcriptional level can reflect some aspects of immune status, but not global changes. Moreover, another independent cohort and *in vitro* or *in vivo* studies should be performed to validate our results.

In conclusion, we hope our results provide novel insights to assist in the design of new immunotherapeutic drugs, to help clinicians choose appropriate drugs for their RCC patients and prognostic biomarkers, and to identify biomarkers to more accurately predict the survival of patients with RCC.

## Data Availability Statement

The datasets analyzed for this study can be found in the Oncomine, GEPIA, UALCAN and cBioPortal web resources, and requests to further access to datasets can be directed to zwlord@outlook.com.

## Author Contributions

ZL and HL performed data analysis work and aided in writing the manuscript. QZ and SS designed the study and assisted in writing the manuscript. YL and XL edited the manuscript. All authors read and approved the final manuscript.

### Conflict of Interest

The authors declare that the research was conducted in the absence of any commercial or financial relationships that could be construed as a potential conflict of interest.

## Abbreviations

RCC: renal cell carcinoma
TCGA: The Cancer Genome Atlas
PPI: protein–protein interaction
GO: Gene Ontology
KEGG: Kyoto Encyclopedia of Genes and Genomes
BP: biological processes
CC: cellular components
MF: molecular function
GSEA: Gene Set Enrichment Analysis.
