# Supplementary material for: Identification of Therapeutic Targets and Prognostic Biomarkers Among CXC Chemokines in the Renal Cell Carcinoma Microenvironment
Source: Front Oncol. 2020 Feb 5;9:1555. doi: 10.3389/fonc.2019.01555 (PMC7012904; doi:10.3389/fonc.2019.01555)
Supplement: Supplementary file 1 [file Image_1.pdf]

Supplementary Material

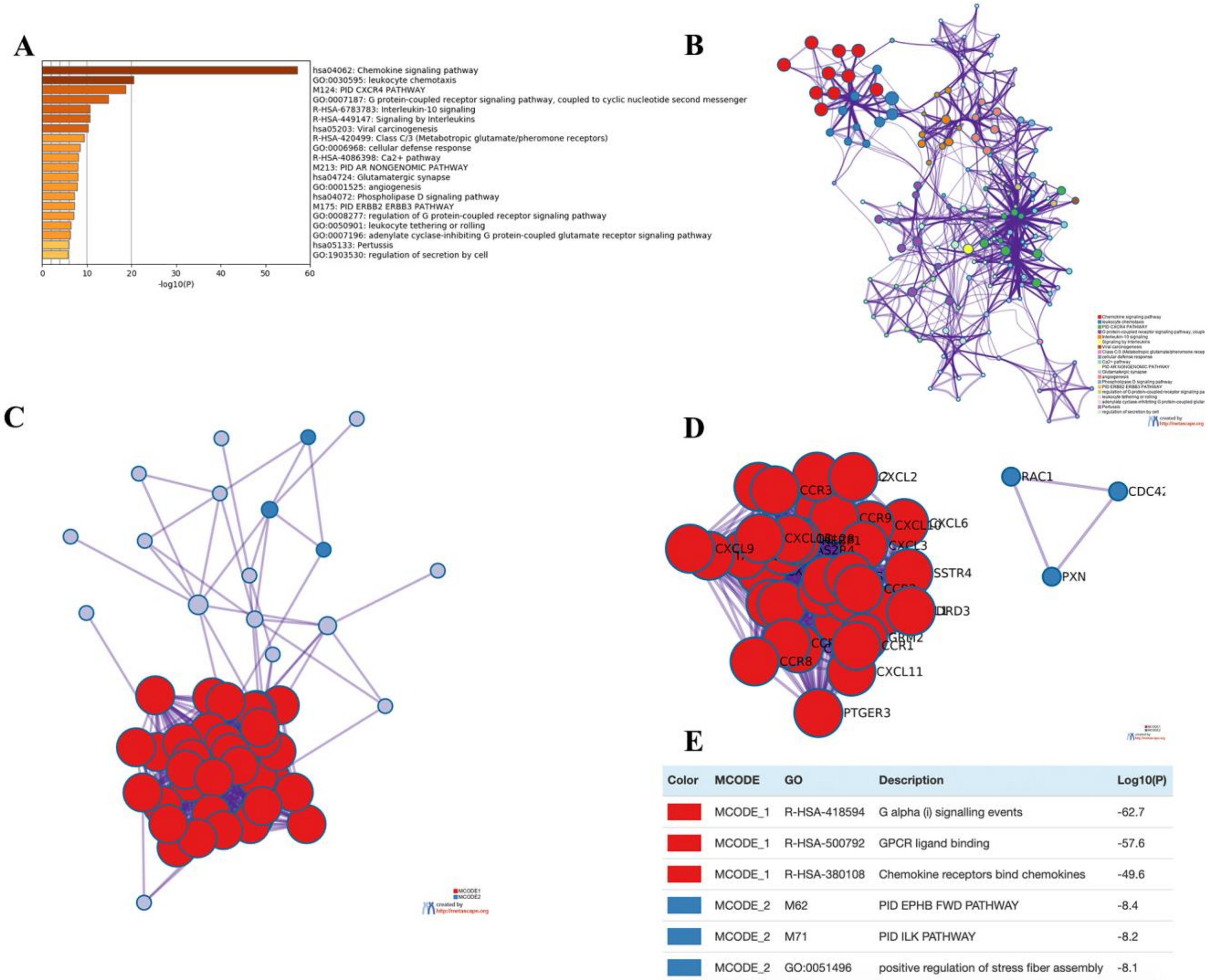

E

Color	MCODE	GO	Description	Log10(P)
	MCODE_1	R-HSA-418594	G alpha (i) signalling events	-62.7
	MCODE_1	R-HSA-500792	GPCR ligand binding	-57.6
	MCODE_1	R-HSA-380108	Chemokine receptors bind chemokines	-49.6
	MCODE_2	M62	PID EPHB FWD PATHWAY	-8.4
	MCODE_2	M71	PID ILK PATHWAY	-8.2
	MCODE_2	GO:0051496	positive regulation of stress fiber assembly	-8.1

Supplementary Figure 1. The enrichment analysis of different expressed CXC chemokines and 50 most frequently altered neighboring genes in RCC (Metascape). (A) Bar graph of top 20 enriched terms across different expressed CXC chemokines and 50 most frequently altered neighboring genes, colored by *p* values. (B) Network of enriched terms, colored by cluster ID. (C, D and E) Protein-protein interaction network and MCODE components identified in different expressed CXC chemokines and 50 most frequently altered neighboring genes.
